# Anthropogenic activities and the regional distribution of leprosy in Senegal: an ecological analysis of agricultural and mining activities

**DOI:** 10.1016/j.ijregi.2026.101043

**Published:** 2026-10-23

**Authors:** Anne marie rose arcens Ndiaye, Cheikh Sokhna, Ghiles Grine

**Affiliations:** 1 Aix-Marseille University, MEPHI, Marseille, France; 2 Aix-Marseille University, SSA, RITMES, Marseille, France; 3 Aix-Marseille University, IRD, SSA, MINES, Marseille, France; 4 IHU Méditerranée Infection, Marseille, France

**Keywords:** Leprosy, *Mycobacterium leprae*, Anthropization, Environmental transmission, Agriculture, Senegal

## Abstract

**Objectives:**

To investigate the association between anthropogenic environmental changes, particularly agricultural and mining activities, and the spatial distribution of leprosy in Senegal.

**Methods:**

An ecological study was conducted across the 14 administrative regions using national surveillance data and cases detected through active case-finding (2020-2022). Agricultural intensity was scored from 0 (urban) to 4 (very high), and mining activity was classified by regional intensity. Associations with leprosy case counts were assessed using correlation, analysis of variance, and linear regression.

**Results:**

Agricultural intensity was strongly associated with regional leprosy case counts (Spearman’s ρ = 0.915, *P* < 0.001). Linear regression explained 48.6% of case-count variation (*R*² = 0.486, *P* = 0.006), with each unit increase in agricultural intensity associated with 81.85 additional cases. The Bassin Arachidier accounted for 68.2% of cases across four of 14 regions. Mining activity was also positively associated with leprosy cases (ρ = 0.647, *P* = 0.012), with significant differences across mining levels (analysis of variance: *F*₂,₁₁ = 4.23, *P* = 0.041).

**Conclusion:**

Higher agricultural and mining intensity were associated with greater regional leprosy case counts. These ecological findings support further investigation of anthropogenic environmental factors in leprosy distribution.

## Highlights

• Leprosy clustered in Senegal's Bassin Arachidier region.
• Over 65% of cases occurred in only four of 14 regions.
• Leprosy hotspots overlapped with farming and mining areas.
• Environmental disturbance may favor *Mycobacterium leprae* persistence.
• Findings support an environmental component of leprosy transmission.

## Introduction

Leprosy, caused by *Mycobacterium leprae*, remains a persistent public health concern in many parts of the world [1,2]. In Senegal, recent observations point to a troubling increase in lepromatous leprosy [3,4]. Over the past decade, Senegal has recorded an average of 15.4 new leprosy cases per million population annually, with a decline to 10.6 cases per million in 2020 [5]. Despite this apparent decrease, leprosy remains underdetected in the country, as evidenced by the identification of numerous new cases during recent active screening campaigns. Although leprosy was officially eliminated as a public health problem in Senegal in 1995, several regions have seen a resurgence in new cases in recent years [6,7]. In response, the National Leprosy Control Program, in collaboration with its partners, carried out a prevalence study in 2016 in two of the nine Social Rehabilitation Villages (SRVs), Koutal and Mballing, originally established in 1976 for the isolation of individuals affected by leprosy. This resurgence appears to be occurring in parallel with ongoing anthropization, the human-driven transformation of natural landscapes into urban, agricultural, or industrial zones.

Anthropization, through processes such as deforestation, urban sprawl, rural exodus, and changes in land use, can profoundly disrupt ecological balances. These environmental transformations are increasingly recognized as potential contributors to the persistence and transmission of *M. leprae*. For instance, the destruction of natural habitats and the expansion of agricultural or urban zones have been linked to increased human contact with known zoonotic reservoirs of *M. leprae*, such as armadillos (*Dasypus novemcinctus*), particularly in the Americas [8,9]. In India, *M. leprae* deoxyribonucleic acid has been detected in the soil surrounding the homes of patients with multibacillary leprosy, suggesting that contaminated environments, often created or modified by human activity, may act as environmental reservoirs [10,11]. These findings underscore the hypothesis that anthropogenic landscapes can sustain *M. leprae* outside human hosts and may play a role in indirect transmission pathways.

The objective of this study was to assess, across the 14 administrative regions of Senegal, the association between regional leprosy case counts and two major anthropogenic activities—agriculture and mining—using national surveillance data complemented by active case-finding data collected between 2020 and 2022. Specifically, we evaluated whether agricultural intensity, agro-ecological characteristics, and mining activity were associated with the regional distribution of leprosy cases.

## Methods

### Study design, setting and data sources

We conducted an ecological observational study using secondary data to explore the association between the spatial distribution of leprosy cases and agricultural and mining activities in Senegal. Senegal is administratively divided into 14 regions, all of which were included in the study and constituted the geographical units of analysis.

Leprosy data were compiled from two complementary sources. First, we extracted leprosy-related data from the 2020 edition of the *Annuaire des statistiques sanitaires et sociales du Sénégal*, covering the 14 administrative regions of the country. These data were complemented with findings from the active case-finding study reported by Batista et al. [3], conducted between October 2020 and February 2022 in nine SRVs. SRVs are specialized settlements established to accommodate and support people affected by leprosy, particularly individuals who have experienced long-term social exclusion related to the disease, visible disabilities, or advanced-stage disease.

The study by Batista et al. [3] involved active door-to-door screening for leprosy and the administration of single-dose rifampicin as post-exposure prophylaxis to eligible household and social contacts within the SRVs. Cases identified through this active case-finding campaign were used to complement the data obtained from the national statistical yearbook for the corresponding regions. Duplicate cases between the two sources were excluded to avoid double counting. The resulting dataset was organized at the regional level, with the 14 administrative regions constituting the units of analysis. The regional distribution of leprosy cases is summarized in Table 1. No population standardization was applied; therefore, the analyses were based on absolute numbers of leprosy cases rather than population-standardized incidence rates.

**Table 1:** Regional distribution of leprosy cases in Senegal by agricultural zone and primary economic activity.

Administrative region	Leprosy cases	Agricultural zone currently indicated	Primary activity	Intensity level
Diourbel	553	Bassin Arachidier	Groundnut cultivation	Very high
Kaolack	190	Bassin Arachidier	Groundnut cultivation	Very high
Kédougou	152	Senegal Oriental	Agroforestry, gold mining	Medium
Saint-Louis	75	Vallée Fleuve/Ferlo	Rice irrigation + livestock	High
Matam	64	Vallée Fleuve	Rice irrigation	High
Thiès	55	Zone côtière/Bassin Arachidier	Market gardening	Medium
Kaffrine	55	Bassin Arachidier	Groundnut cultivation	High
Fatick	36	Zone côtière	Mixed agriculture	Medium
Louga	21	Ferlo	Extensive livestock	Low
Tambacounda	14	Senegal Oriental	Extensive agriculture	Low
Kolda	6	Casamance	Rice cultivation	Low-medium
Sédhiou	2	Casamance	Rice cultivation	Low
Dakar	0	Zone côtière	Urban/periurban	Urban
Ziguinchor	0	Casamance	Rice cultivation	Low

Confirmed corrections: Diourbel = 553 cases; Thiès = 55 total cases. Thiès is not split into 35/20 because no subregional case allocation was provided. Agro-ecological classifications are retained as currently indicated and have not yet been harmonized.

To characterize the socio-environmental context of leprosy distribution, we additionally collected regional information on major agricultural activities and geospatial information on the distribution of mining and mineral resources across Senegal.

Agricultural activities were characterized by using a multidimensional classification system. The 14 administrative regions were characterized according to their predominant agro-ecological zone and farming system. Because administrative and agro-ecological boundaries do not always coincide, some regions may overlap more than one agro-ecological zone. For the regional-level analysis, each administrative region was considered as a single epidemiological unit, and regional leprosy case counts were not subdivided between agro-ecological zones (Table 2): 0 = urban, 1 = low intensity, 2 = medium intensity, 3 = high intensity, and 4 = very high intensity. The predominant agricultural activity in each region was also recorded as a categorical variable.

**Table 2:** Regional distribution of leprosy cases according to agro-ecological characteristics, agricultural intensity, and mining activity in Senegal.

Administrative region	Leprosy cases	Agro-ecological group	Predominant agricultural activity	Agricultural intensity category	Intensity score (0-4)
Diourbel	553	Bassin Arachidier	Groundnut cultivation	Very high	4
Kaolack	190	Bassin Arachidier	Groundnut cultivation	Very high	4
Kédougou	152	Senegal Oriental	Agroforestry, gold mining	Medium	2
Saint-Louis	75	Vallée du Fleuve/Ferlo	Rice irrigation + livestock	High	3
Matam	64	Vallée du Fleuve/Ferlo	Rice irrigation	High	3
Thiès	55	Niayes	Market gardening	Medium	2
Kaffrine	55	Bassin Arachidier	Groundnut cultivation	High	3
Fatick	36	Bassin Arachidier	Mixed agriculture	Medium	2
Louga	21	Vallée du Fleuve/Ferlo	Extensive livestock	Low	1
Tambacounda	14	Senegal Oriental	Extensive agriculture	Low	1
Kolda	6	Casamance	Rice cultivation	Low	1
Sédhiou	2	Casamance	Rice cultivation	Low	1
Dakar	0	Niayes	Urban/periurban	Urban	0
Ziguinchor	0	Casamance	Rice cultivation	Low	1
**Mining activity summary**					
**Mining activity category**	**Number of regions (n)**	**Mean leprosy cases**	**SD**		
High	4	208.3	238.4		
Medium	5	63.2	67.8		
Low	5	14.2	25.4		
**Agricultural intensity scoring key**					
0 = Urban	1 = Low	2 = Medium	3 = High	4 = Very high	

Mining activity data were obtained from the 2021 database of the Initiative pour la Transparence dans les Industries Extractives, which provides spatial information on mineral resources and mining sites in Senegal, including gold, phosphate, iron ore, and other mineral resources. Based on visual assessment of the spatial distribution of mineral resources represented on the Initiative pour la Transparence dans les Industries Extractives maps, the 14 administrative regions were classified into four categories: high, medium, low, or no mining activity.

## Analyses

### Statistical analysis

#### Descriptive statistics

Summary statistics including mean, median, standard deviation (SD), minimum, and maximum values were calculated for leprosy cases by agricultural zone, intensity level, and mining activity category.

#### Correlation analysis

Both parametric and non-parametric correlation analyses were performed: Spearman's rank correlation coefficient (ρ) to assess monotonic relationships and Pearson's correlation coefficient (r) for linear relationship assessment.

#### Comparative analysis

Between-group differences were evaluated using (1) one-way analysis of variance (ANOVA) for normally distributed data and (2) the Kruskal–Wallis test as a non-parametric alternative. Effect sizes were calculated using eta-squared (η²).

#### Regression analysis

Linear regression was performed to model the relationship between agricultural intensity and leprosy cases: model specification: leprosy cases = β₀ + β₁(agricultural intensity). Model diagnostics included *R*², adjusted *R*², F statistic, and root mean square error.

#### Post hoc analysis

Tukey's honestly significant difference test was conducted for pairwise comparisons following significant ANOVA results.

#### Statistical software and significance level

All analyses were performed using standard statistical software RStudio version 2026.10.1 (Posit Software, PBC, Boston, MA, USA) [12], with the significance level set at α = 0.05. Results are presented with 95% confidence intervals where applicable.

### Ethical considerations

The present ecological study was based exclusively on the secondary analysis of aggregated data obtained from previously published and publicly available sources. No participants were directly recruited for the present study, and no identifiable individual-level data were collected or analyzed.

## Results

The ecological analysis included all 14 administrative regions of Senegal, which constituted the geographical units of analysis. For the assessment of agricultural and environmental characteristics, the 14 administrative regions were characterized according to their predominant agro-ecological zone and agricultural activity. Regional leprosy case counts were retained at the administrative-region level without subdivision between overlapping agro-ecological zones. The distribution of leprosy cases was examined in relation to agro-ecological zone, agricultural intensity, and mining activity. The results showed substantial geographical variation in regional leprosy case counts, as well as positive associations between leprosy case counts and the agricultural and mining characteristics investigated.

### Association between agricultural intensity and leprosy cases

In addition to the geographical differences observed across agro-ecological groups, agricultural intensity was positively associated with regional leprosy case counts. Spearman's rank correlation showed a strong positive monotonic association between agricultural intensity score and leprosy case counts (ρ = 0.915, *P* < 0.001) (Figure 1). Thus, regions characterized by higher agricultural intensity scores tended to have higher numbers of recorded leprosy cases.

**Figure 1:**
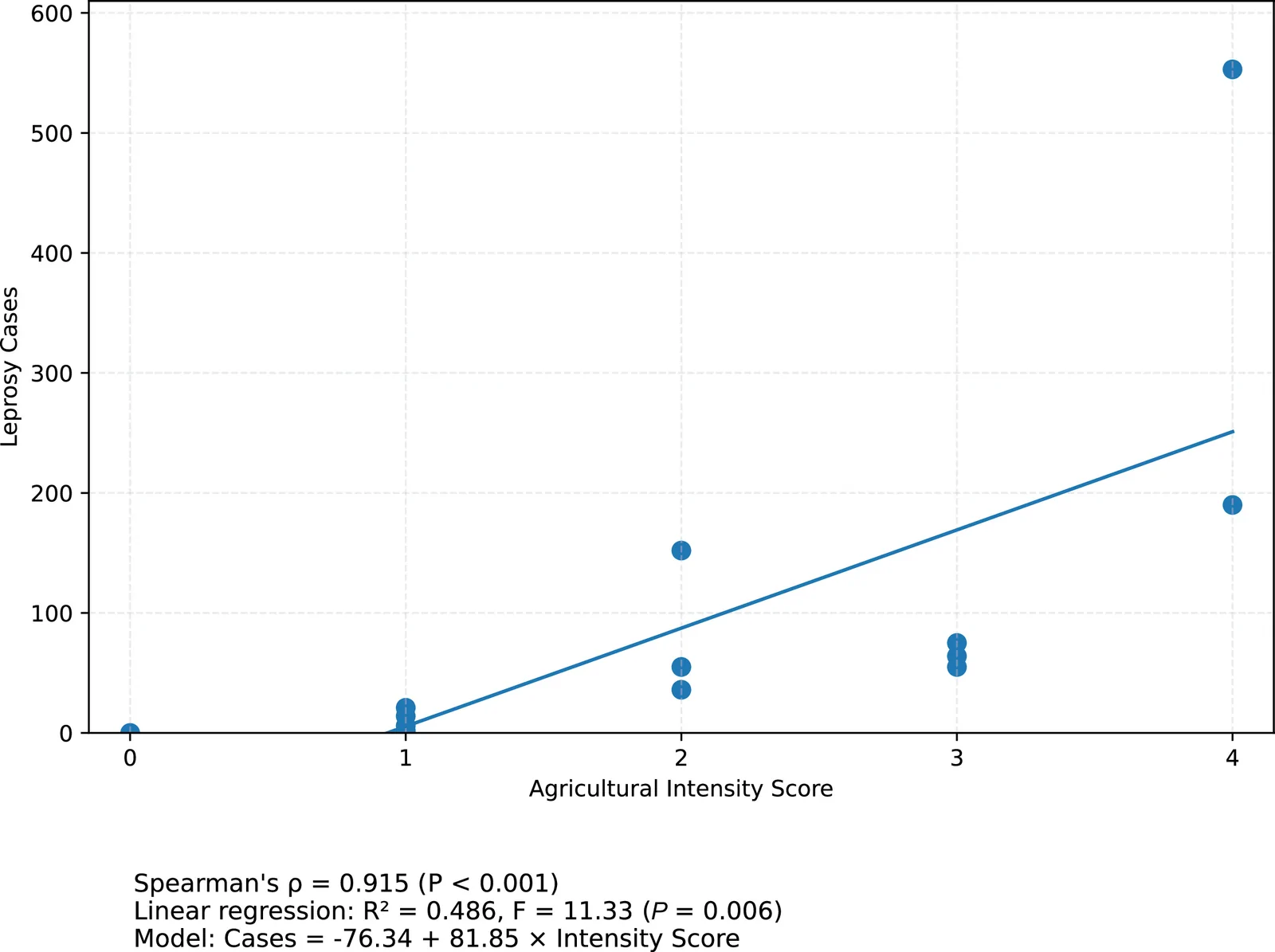
Agricultural intensity vs leprosy cases.

This relationship was further explored using simple linear regression. The regression model showed a positive association between agricultural intensity score and regional leprosy case counts, with the fitted equation: predicted leprosy cases = −76.34 + 81.85 × agricultural intensity score. The model explained 48.6% of the observed variation in regional leprosy case counts (*R*² = 0.486) and was statistically significant (*F* = 11.33, *P* = 0.006). Accordingly, each 1-unit increase in agricultural intensity score was associated with an estimated increase of 81.85 leprosy cases (Figure 1).

### Differences in leprosy cases across agro-ecological zones

Differences in leprosy case counts across the five agro-ecological groups were evaluated statistically. One-way ANOVA did not identify a statistically significant difference between groups (*F*₄,₉ = 1.11, *P* = 0.411), with an eta-squared value of 0.329. Similarly, the Kruskal–Wallis test did not show a statistically significant difference across agro-ecological groups (*H* = 7.21, *df* = 4, *P* = 0.125).

### Agricultural activities and regional distribution of leprosy cases

The distribution of leprosy cases varied across the five agro-ecological groups (Table 2; Figure 2). The Bassin Arachidier, comprising four regions, showed the highest mean number of leprosy cases (208.5 ± 239.7 (SD) cases). This was followed by Senegal Oriental, comprising two regions, with a mean of 83.0 ± 97.6 (SD) cases. The three regions classified within Vallée du Fleuve/Ferlo had a mean of 53.3 ± 28.5 (SD)cases. The Niayes group, comprising two regions, had a mean of 27.5 ± 38.9 cases, while Casamance, comprising three regions, showed the lowest mean number of cases (2.7 ± 3.1 (SD)). These descriptive findings indicate substantial geographical heterogeneity in regional leprosy case counts across the agro-ecological groups.

**Figure 2:**
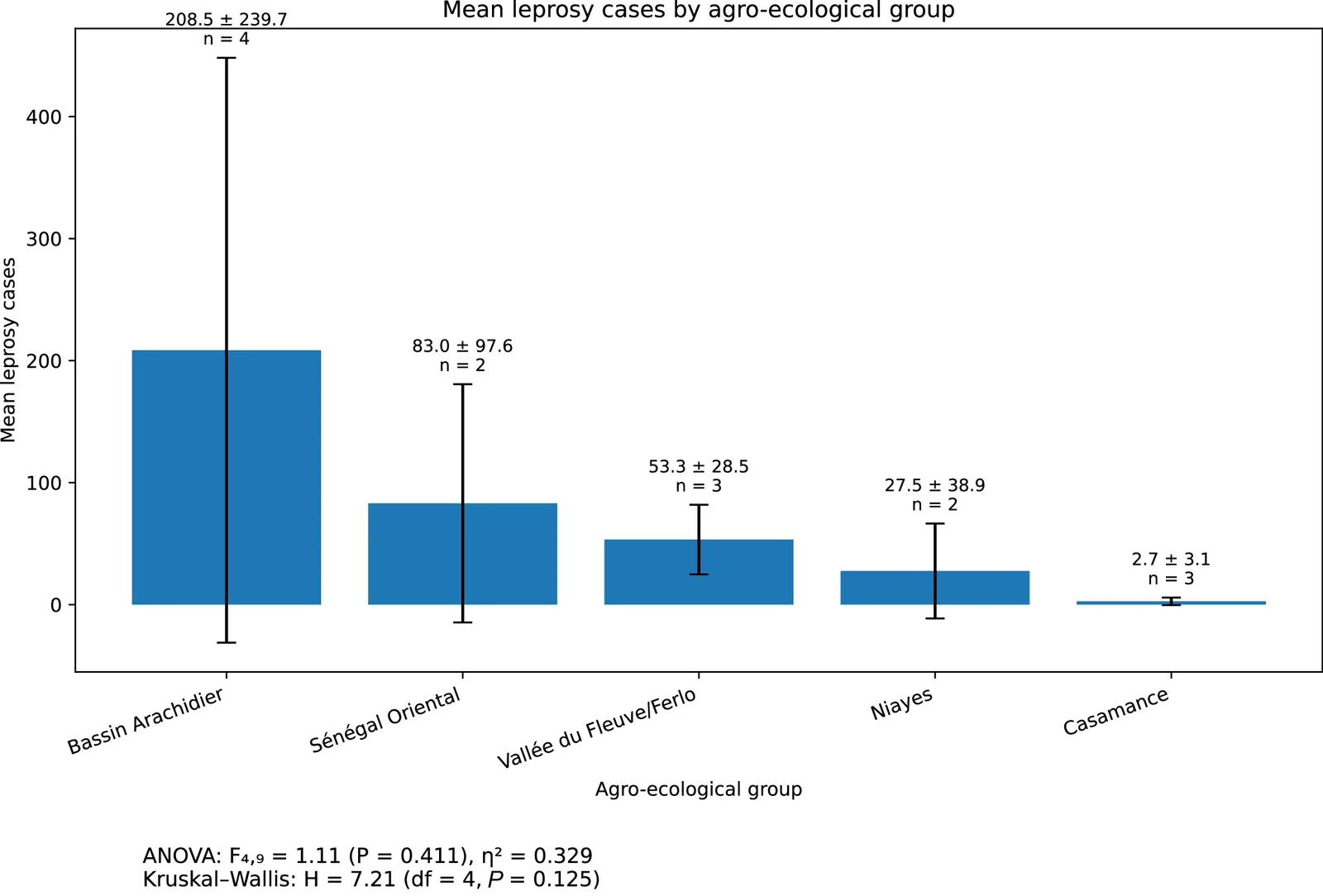
Mean leprosy cases by agricultural zone.

### Mining activity and regional distribution of leprosy cases

Regional leprosy case counts also varied according to the level of mining activity (Table 2). Among the 14 regions included in the ecological analysis, four were classified as having high mining activity, five as having medium mining activity, and five as having low mining activity. Regions classified as having high mining activity had a mean of 208.3 leprosy cases (SD = 238.4). In comparison, regions with medium mining activity had a mean of 63.2 cases (SD = 67.8), while regions with low mining activity had a mean of 14.2 cases (SD = 25.4). The descriptive results therefore showed progressively higher mean leprosy case counts across the reported mining activity categories, from low to high activity.

Correlation analyses were consistent with this pattern. Spearman's rank correlation showed a positive association between mining activity level and regional leprosy case counts (ρ = 0.647, *P* = 0.012). Pearson's correlation analysis also indicated a positive relationship (*r* = 0.592, *P* = 0.026). These results indicate that regions classified at higher levels of mining activity tended to have higher recorded numbers of leprosy cases.

Differences between mining activity categories were further assessed using one-way ANOVA. The analysis indicated differences in leprosy case counts across mining activity levels (*F*₂,₁₁ = 4.23, *P* = 0.041), with an eta-squared value of 0.435. *Post hoc* analysis showed a difference between regions classified as having high mining activity and those classified as having low mining activity, with a mean difference of 194.1 cases (95% confidence interval: 8.2-380.0, *P* = 0.038) (Figure 3).

**Figure 3:**
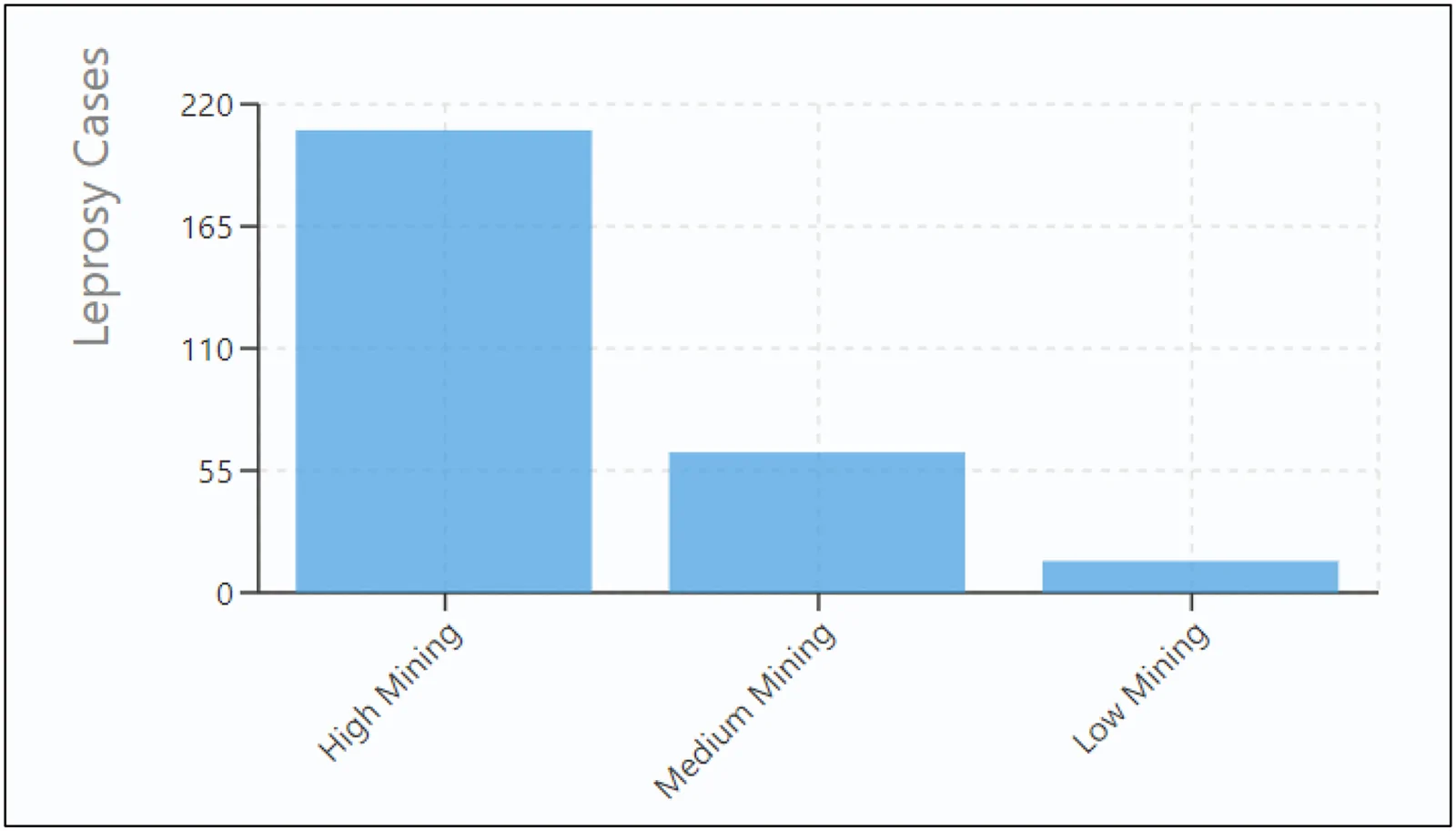
Mean leprosy cases by mining activity level. Abbreviations: ANOVA, analysis of variance; CI, confidence interval.

Taken together, the results showed substantial regional heterogeneity in leprosy case counts across Senegal. Higher agricultural intensity and higher categories of mining activity were both positively associated with regional leprosy case counts, while marked differences were also observed among agro-ecological zones.

## Discussion and conclusion

This ecological analysis revealed significant positive associations between both agricultural and mining activities and leprosy case distribution across Senegal's regions. The agricultural intensity analysis demonstrated particularly robust correlations, with very high-intensity agricultural regions showing dramatically elevated case numbers compared to low-intensity areas. The strong correlation between agricultural intensity and leprosy cases (Spearman’s ρ = 0.915, *P* < 0.001) suggests that intensive farming practices may create environmental conditions conducive to *M. leprae* transmission or survival. Recent studies have demonstrated the presence of *M. leprae* deoxyribonucleic acid in soil samples from leprosy-endemic regions, indicating that environmental sources could play a role as a reservoir for transmission [13]. The detection of viable *M. leprae* in environmental samples, coupled with genetic profiling showing identical strain types in both patients and environmental sources, suggests that both environment and patients contribute to disease transmission [14]. The Bassin Arachidier, characterized by intensive groundnut cultivation, accounted for 68.2% of total leprosy cases despite representing only three of 14 regions. This pattern may reflect several potential mechanisms: enhanced soil disturbance, increasing mycobacterial aerosolization, specific agricultural practices creating favorable environmental niches for the pathogen, or socioeconomic factors associated with agricultural labor. Environmental factors, particularly humidity, may favor *M. leprae* transmission and survival in agricultural settings [15]. Given the ecological design of the study, this association should not be interpreted as evidence of a causal relationship or individual-level risk.

The anomalously low case numbers in Casamance regions, despite active rice cultivation, suggest that not all agricultural activities carry equivalent risk. This variation may relate to differences in farming techniques, water management practices, or regional environmental factors affecting mycobacterial survival and transmission. *M. leprae* has been detected in various environmental matrices including soil, water, and plants, with survival dependent on specific environmental conditions [16].

The moderate correlation between mining intensity and leprosy cases (Spearman’s ρ = 0.647, *P* = 0.012) indicates another potential pathway for environmental exposure. Mining activities may facilitate mycobacterial transmission through soil disruption, dust generation, or creation of specific microenvironmental conditions. The significant difference between high and low mining activity regions supports this hypothesis, though the mechanisms remain speculative.

Notably, this environmental-occupational disease association parallels findings from studies of Buruli ulcer, another mycobacterial infection caused by *Mycobacterium ulcerans*. Research in Benin and Guinea has demonstrated significant associations between Buruli ulcer incidence and environmental disturbances, particularly mining operations, deforestation, and agriculture [17]. Gold-mining regions specifically show increased Buruli ulcer incidence, with changes in water quality due to mining activities contributing to disease emergence [18,19]. Like leprosy, Buruli ulcer represents an ecosystem-related infection where skin contamination combined with environmental exposure constitutes the most plausible transmission route [20]. This parallel suggests that mycobacterial diseases may share common environmental transmission pathways linked to anthropogenic activities that disturb natural ecosystems.

Several important limitations constrain the interpretation of these findings. The limited number of geographical units included in the ecological analysis (*n* = 14 administrative regions) limits statistical power and generalizability. The absence of population-standardized incidence rates prevents assessment of true epidemiological risk. Potential confounding variables including population density, socioeconomic status, healthcare access, and regional migration patterns were not controlled. The cross-sectional design precludes causal inference, and temporal factors regarding the duration of exposure and disease latency were not considered.

These findings suggest that intensive agriculture and mining-related environmental disturbance may be associated with the regional distribution of leprosy cases in Senegal. If confirmed through controlled studies, these associations could inform targeted surveillance and prevention strategies in high-risk occupational settings. The parallel with Buruli ulcer reinforces the importance of considering environmental mycobacterial reservoirs in the epidemiology of both diseases, particularly in contexts of anthropogenic environmental disturbance [21].

As a testable mechanistic hypothesis, we propose that *M. leprae* may persist in a dormant state within soil environments characterized by oxygen-depleted conditions. Under such anaerobic or hypoxic conditions, the bacilli could remain in a low-metabolic or dormant state. However, human activities, particularly mining and agricultural operations, disturb these oxygen-poor soil layers, bringing dormant *M. leprae* into contact with atmospheric oxygen. We hypothesize that subsequent exposure to more oxygenated conditions could favor bacterial reactivation, transitioning the pathogen from a dormant state toward a more metabolically active form. This proposed mechanism is not demonstrated by the present ecological analysis and requires experimental validation.

Mining activities, through excavation and soil displacement, and agricultural practices, including plowing, tilling, and irrigation, effectively aerate previously oxygen-depleted soil strata. Such oxygenation could potentially create conditions favoring *M. leprae* reactivation and thereby increase opportunities for environmental exposure. If experimentally confirmed, this mechanism could contribute to explaining the geographical patterns observed in regions with intensive agricultural development or mining operations, where soil disturbance is more pronounced.

As a conclusion, this study identified positive associations between agricultural intensity, mining activity, and the regional distribution of leprosy cases in Senegal. These findings support further investigation of environmental and occupational factors that may contribute to the persistence of leprosy in endemic settings. Because the analysis was conducted at the regional level using absolute case counts, the observed associations cannot establish causality or individual-level risk. Population-standardized, longitudinal, and experimental studies are needed to confirm these observations and to investigate the proposed environmental mechanisms.

## Funding

This work was supported by the French Government through the French National Research Agency (ANR) under the “Investissements d'avenir” (Investments for the Future) programme (grant ANR-10-IAHU-03, Méditerranée Infection), the Contrat de Plan État-Région (CPER), and the European Regional Development Fund (ERDF/FEDER) through the IHUPERF programme.

## CRediT authorship contribution statement

**Anne marie rose arcens Ndiaye:** Writing – review & editing, Writing – original draft, Visualization, Methodology, Conceptualization. **Cheikh Sokhna:** Writing – original draft, Visualization, Supervision, Data curation, Conceptualization. **Ghiles Grine:** Writing – review & editing, Writing – original draft, Visualization, Validation, Supervision, Resources, Methodology, Investigation, Formal analysis, Data curation, Conceptualization.

## Declaration of competing interest

The authors have no competing interests to declare.
